# Mangiferin Prevents Guinea Pig Tracheal Contraction via Activation of the Nitric Oxide-Cyclic GMP Pathway

**DOI:** 10.1371/journal.pone.0071759

**Published:** 2013-08-08

**Authors:** Aline B. Vieira, Luciana P. Coelho, Daniella B. R. Insuela, Vinicius F. Carvalho, Marcelo H. dos Santos, Patricia MR. Silva, Marco A. Martins

**Affiliations:** 1 Laboratory of Inflammation, Oswaldo Cruz Institute, Oswaldo Cruz Foundation, Rio de Janeiro, RJ, Brazil; 2 Laboratory of Phytochemistry and Medicinal and Chemistry, Department of Pharmacy, Alfenas, Federal University of Alfenas, MG, Brazil; Faculté de médecine de Nantes, France

## Abstract

Previous studies have described the antispasmodic effect of mangiferin, a natural glucoside xanthone (2-C-β-Dgluco-pyranosyl-1,3,6,7-tetrahydroxyxanthone) that is present in mango trees and other plants, but its mechanism of action remains unknown. The aim of this study was to examine the potential contribution of the nitric oxide-cyclic GMP pathway to the antispasmodic effect of mangiferin on isolated tracheal rings preparations. The functional effect of mangiferin on allergic and non-allergic contraction of guinea pig tracheal rings was assessed in conventional organ baths. Cultured tracheal rings were exposed to mangiferin or vehicle, and nitric oxide synthase (NOS) 3 and cyclic GMP (cGMP) levels were quantified using western blotting and enzyme immunoassays, respectively. Mangiferin (0.1–10 µM) inhibited tracheal contractions induced by distinct stimuli, such as allergen, histamine, 5-hydroxytryptamine or carbachol, in a concentration-dependent manner. Mangiferin also caused marked relaxation of tracheal rings that were precontracted by carbachol, suggesting that it has both anti-contraction and relaxant properties that are prevented by removing the epithelium. The effect of mangiferin was inhibited by the nitric oxide synthase inhibitor, Nω-nitro-L-arginine methyl ester (L-NAME) (100 µM), and the soluble guanylate cyclase inhibitor, 1H-[1], [2], [4]oxadiazolo[4,3-a]quinoxalin-1-one (ODQ) (10 µM), but not the adenylate cyclase inhibitor, 9-(tetrahydro-2-furyl)adenine (SQ22536) (100 µM). The antispasmodic effect of mangiferin was also sensitive to K^+^ channel blockers, such as tetraethylammonium (TEA), glibenclamide and apamin. Furthermore, mangiferin inhibited Ca^2+^-induced contractions in K^+^ (60 mM)-depolarised tracheal rings preparations. In addition, mangiferin increased NOS3 protein levels and cGMP intracellular levels in cultured tracheal rings. Finally, mangiferin-induced increase in cGMP levels was abrogated by co-incubation with either ODQ or L-NAME. These data suggest that the antispasmodic effect of mangiferin is mediated by epithelium-nitric oxide- and cGMP-dependent mechanisms.

## Introduction

The xanthone mangiferin is an active phytochemical compound with therapeutic potential that is primarily found in mango tree leaves and stem bark (*Mangifera indica*) [1], [2]. This substance usually occurs as a glucoside and is also found in a variety of other plant families, including *Anemmarhena asphodeloides* [3], *Bersama abyssinica* [4], *Cyclopia genistoides* [5], *Cyclopia subternata* [6], *Gentiana lutea* [7], *Gnidia involucrata* [8], *Rhizoma belamcandae* [9] and *Salacia oblonga* [10], among others. Notably, *M. indica* and several of the above listed plants have been traditionally used to treat important human diseases, such as diabetes [10], obesity [10], cancer [11], and asthma [12].

An extract obtained via the decoction and drying of mango stem bark was developed at industrial scale in Cuba for use as a nutritional supplement and phytomedicine [13]. Vimang® is the brand name of this commercial preparation, and it contains a standardised mixture of terpenoids, steroids, fatty acids and polyphenols, including phenolic acids, phenolic esters and the predominant component mangiferin [13].

Similar to other polyphenol compounds, such as anthocyanins, curcumin and resveratrol, mangiferin has a broad spectrum of pharmacological effects. The most prominent and best-studied property of this class of phytochemicals is their antioxidant activity [1], [14]. The ability to scavenge and decrease the formation of reactive oxygen species, as well as to activate enzymatic antioxidant systems, seems to be crucial for the outstanding antioxidant activity of mangiferin [1], [14], [15]. Apart from its capacity to interfere with oxidative stress, mangiferin exhibits a number of other properties, including immune-modulatory [16]–[18], anti-inflammatory [19]–[21] and anti-cancer [11], [22], [23] activities, suggesting that this substance could be used as a molecular template for innovative therapeutic applications.

The free radical nitric oxide is a neurotransmitter of the inhibitory nonadrenergic noncholinergic respiratory system [24], [25]. It is produced by neural fibres that innervate airway smooth muscle cells, epithelial ciliated cells, type II alveolar cells and macrophages, and nitric oxide has been described as an effective antispasmodic mediator in the airway [26]. The molecular mechanism underlying the antispasmodic effect of nitric oxide is the direct activation of soluble guanylate cyclase and subsequent elevation of intracellular cGMP levels [27].

The aim of the present study was to assess the potential protective effect of mangiferin on the contractile response presented by the rat tracheal smooth muscle, following exposure to distinct pro-spasmodic agents, such as histamine, 5-hydroxytryptamine (5-HT), carbachol and allergen *in vitro*. All these spasmogens are supposed to play important role in the pathogenesis of airway obstruction noted in atopic asthmatics. Indeed, earlier investigations have shown that *M. indica* stem bark aqueous extract is an effective inhibitor of rat tracheal contraction caused by acetylcholine [28], [29] and histamine [29]. However, exactly how the extract is acting to induce anti-contraction effects and whether or not this effect is accounted for by mangiferin has not been studied. Furthermore, our intention with this study was to test the hypothesis that mangiferin might be acting as an antispasmodic agent via activation of the nitric oxide-cGMP pathway. The results show that mangiferin can indeed inhibit smooth muscle spasms triggered by immunological and non-immunological stimuli. Such an effect is associated with nitric oxide production by epithelial cells, up-regulation of intracellular cGMP and the opening of K^+^_ATP_ and small-conductance Ca^2+^-activated K^+^ channels in airway smooth muscle cells.

## Methods

### Ethics Statement

Experimental conditions and procedures involving animals were performed with direct approval of the Committee on Use of Laboratory Animals of the Oswaldo Cruz Foundation under license no. CEUA-FIOCRUZ 00085-01.

### Animals

Male guinea pigs (300–400 g) were obtained from the Oswaldo Cruz Foundation breeding unit (Rio de Janeiro, Brazil). They were housed under conditions of constant temperature and controlled illumination, and food and water were available ad libitum.

### Drugs and Chemical Reagents

Sodium chloride (NaCl), potassium chloride (KCl), potassium dihydrogen phosphate (KH_2_PO_4_), sodium hydrogen carbonate (NaHCO_3_), magnesium sulfate heptahydrate (MgSO_4_ • 7H_2_O), calcium chloride dihydrate (CaCl_2_ • 2H_2_O) and dimethyl sulfoxide (DMSO) were purchased from Merck (Darmstadt, Germany). Mangiferin, Glucose, EGTA, histamine, 5-hydroxytryptamine (5-HT), ovalbumin, carbachol, N-nitro-L-arginine methyl ester (L-NAME), tetraethylammonium (TEA), glibenclamide, apamin and 1H-[1], [2], [4]oxadiazolo[4,3-a]quinoxalin-1-one (ODQ) were purchased from Sigma-Aldrich (St. Louis, MO). All solutions were freshly prepared in distilled water or DMSO (final concentration, 0.1%) immediately before use.

### Isolated Tracheal Preparation and Measurement of Tension

Guinea pigs used for anaphylactic contraction assays were presensitised with a subcutaneous injection of 0.2 ml of a suspension containing ovalbumin (50 µg) and Al(OH)_3_ (5 mg). The animals were sacrificed in a CO_2_ atmosphere 14 days after sensitisation, and their tracheal segments were removed and quickly immersed in Krebs’ nutritional solution (118 mM NaCl, 4.8 mM KCl, 2.5 mM CaCl_2_, 1.2 mM MgSO_4_, 1.2 mM KH_2_PO_4_, 24 mM NaHCO_3_, and 11 mM glucose). Adhering fat and connective tissue were dissected away from the trachea, and the trachea was then cut into rings. The tracheal rings were mounted in isolated organ baths filled with 10 ml of Krebs’ solution, maintained at 37°C, and aerated with 95% O_2_ and 5% CO_2_. To achieve a steady spontaneous tone level, an initial tension of 1 g was applied. Contractions were measured with an isometric force-displacement transducer (Ugo Basile, Comerio, Italy) and recorded by an Isolated Organs Data Acquisition program (Proto5; Letica Scientific Instruments, Barcelona, Spain).

### Protocols for Measurement of Tension Development

The experimental protocols were previously described [30]. Briefly, the tracheal rings were allowed to stabilise for 60 min, whereas the bathing solution was exchanged at 10 min intervals. At the end of the equilibration period, isolated tracheal rings, in absence or presence of epithelium, were contracted with carbachol (2.5 µM), and once the contractions had reached a plateau, various concentrations of vehicle (DMSO) or mangiferin (0.1–1000 µM) were added. All relaxations are expressed as the percentage of the maximal carbachol-induced contractile responses.

We also investigated the spasmolytic effect of mangiferin on isolated tracheal rings. At the end of the equilibration period, the response to carbachol (2.5 µM) was recorded. After carbachol was washed out and a stable baseline tone was re-established, the tissues were exposed to carbachol (0.01–100 µM), histamine (0.1–1000 µM), 5-HT (0.01–30 µM), or antigen (ovalbumin; 0.001–100 µg/ml) in the presence or absence of mangiferin (0.1–10 µM). The preparations were pre-incubated with mangiferin for 15 min before the addition of each spasmogen. All responses were expressed as a percentage of the initial response to 2.5 µM carbachol. In some experiments, the epithelial cells were removed mechanically by rubbing the internal tracheal surface with a fine silver wire (200 µm in diameter), as described previously [31]. During the experiment, the contractile response to carbachol (0.01–100 µM) was measured before and after exposing intact or denuded epithelium tracheal rings to 10 µM mangiferin for 15 min.

To evaluate the putative interference of mangiferin with calcium influx, Ca^2+^ concentration-response curves were established. Briefly, the responses of tracheal ring segments from naive guinea pig to 2.5 µM carbachol were recorded. After the carbachol was washed out and a stable baseline tone was re-established, the tissues were exposed to successive cycles of 60 mM KCl stimulations/washouts in Ca^2+^-free Krebs’ solution containing 2 mM EGTA until complete desensitisation to the 60 mM KCl-evoked contractile response was achieved. Next, the tracheal rings were immersed in Ca^2+^-free Krebs’ solution containing 60 mM KCl, and the extracellular Ca^2+^ concentration was increased stepwise by the cumulative addition of CaCl_2_ (0.01–30 mM), in the presence or absence of mangiferin (0.1–10 µM) or vehicle (0.1% DMSO). All responses were expressed as a percentage of response to 2.5 µM carbachol.

To further investigate the mechanisms of action of mangiferin, the tracheal rings were pretreated 10 min before mangiferin application with 10 µM ODQ, an inhibitor of guanylate cyclase; 100 µM L-NAME, an inhibitor of NOS; 100 µM SQ22536, an inhibitor of adenylate cyclase; 10 µM TEA, a nonselective K^+^ channel blocker; 1 µM glibenclamide, a K^+^_ATP_ channel blocker or 1 µM apamin, Ca^2+^-dependent K^+^ channel blocker of small conductance. All responses were expressed as a percentage of the response to 2.5 µM carbachol.

### Western Blotting for NOS3

Three pools of three 3 rat tracheas were incubated with 10 µM mangiferin or vehicle (0.1% DMSO) for 15 min., and then homogenized in ice cold lysis buffer containing the protease inhibitor cocktail Complete (F.Hoffmann-La Roche Ltd., Basel, Switzerland) and 0.1% Triton X-100 in PBS. The lysate was centrifuged at 13.000×g for 10 min at 4°C. Supernatant was recovered and protein concentration was determined using the BCA assay (Sigma-Aldrich Corp., St Louis, USA). Equal amounts of sample protein (100 µg/lane) were separated by SDS-PAGE using polyacrylamide gels and proteins were transferred to nitrocellulose membranes (GE Healthcare, Little Chalfont, UK). Nonspecific binding was blocked with 5% (w/v) skimmed milk powder in TTBS for 1 h followed by incubation with rabbit polyclonal antibody NOS3 (1∶ 500; Santa Cruz Biotechnology, CA, USA) or mouse monoclonal antibody β-actin (1∶ 1000; Santa Cruz Biotechnology, CA, USA) overnight at 4°C. Membranes were incubated with HRP-conjugated secondary antibody (1∶ 10.000, R&D Systems, MN, USA ) for 1 h at room temperature. The membranes were washed in TTBS and protein expression was detected using enhanced chemiluminescence (SuperSignal West Dura, Thermo Fisher Scientific Inc., Rockford, USA). Bands intensity was quantified by densitometry (Image-Pro® Plus Media Cybernetics, Bethesda, MD).

### Measurement of cGMP

Intracellular cGMP concentrations in guinea pig tracheal rings were assayed as described previously [30]. Isolated tracheas were cut into rings, quickly immersed in Krebs’ nutritional solution, and incubated with mangiferin (0.1–10 µM), L-NAME (100 µM) or ODQ (10 µM) in the presence of 100 µM 3-isobutyl-1-methylxanthine (IBMX) for 20 min. Some tracheal rings were pretreated with 100 µM L-NAME or 10 µM ODQ for 10 min before the addiction of 10 µM mangiferin. Tissue sections were rapidly frozen in liquid nitrogen, and the frozen tracheal rings were homogenised in ice-cold 6% trichloroacetic acid (TCA). The homogenate was centrifuged at 2000×g for 15 min at 4°C. To remove TCA, the supernatants were washed 4 times with 5 volumes of water-saturated diethyl ether. The top ether layer was discarded after each wash. Then, the supernatants were lyophilised, and the cGMP of each sample was determined using commercially available enzyme immunoassay kits (GE Healthcare, Chalfont St. Giles, UK).

### Statistical Analysis

The results were expressed as the mean ± S.E.M. EC_50_ values were calculated by fitting the log (agonist) *vs.* normalised response using GraphPad Software, and the results are displayed as the negative logarithm (*p*EC_50_). Significant differences were determined using one-way analysis of variance (ANOVA), followed by the Student-Newman-Keuls test. *P* values of 0.05 or less were considered to be statistically significant.

## Results

### Mangiferin Prevents Allergen-, Histamine-, 5-HT- and Carbachol-induced Tracheal Contraction

**Figure 1:**
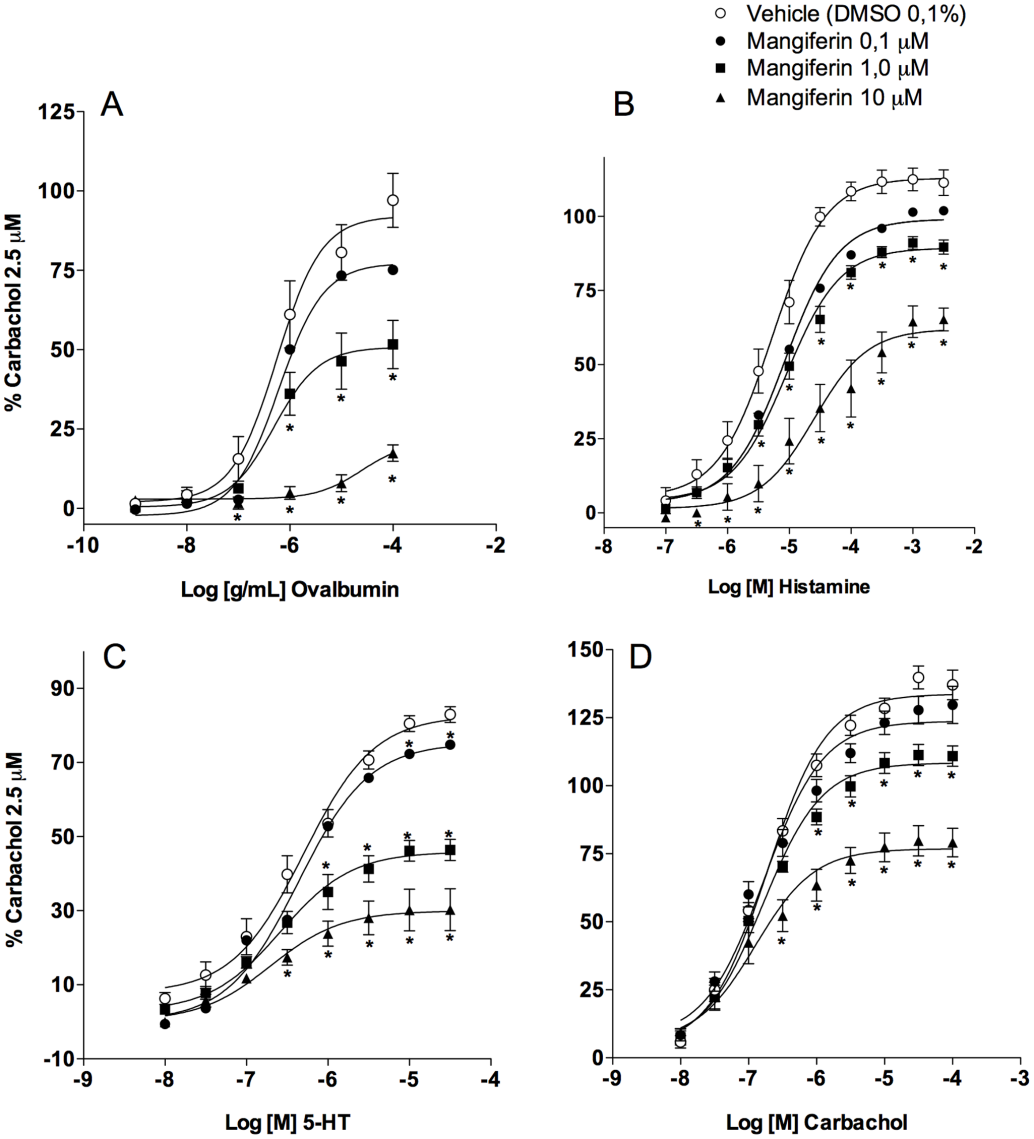
Mangiferin inhibits allergen- and spasmogen-induced tracheal contractions. The antispasmodic effects of mangiferin (0.1–10 µM) on guinea pig tracheal contraction induced by ovalbumin (0.001–100 µg/ml) (A), histamine (0.1–3000 µM) (B), 5-HT (0.01–30 µM) (C) or carbachol (0.01–100 µM) (D). Each point represents the mean ± S.E.M. of 4 to 8 segments. All results are expressed as a percentage of the contractile response induced by 2.5 µM carbachol. * *p*<0.05 compared with the tracheal responses of vehicle-treated tissues (open circles).

It was previously reported that the aqueous extract of *M. indica,* of which mangiferin is the major active constituent [1], inhibited acetylcholine- or histamine-induced rat tracheal contractions [28], [29]. To better characterise the putative antispasmodic property of mangiferin, we first investigated its ability to inhibit the anaphylactic contraction of tracheal rings obtained from ovalbumin-sensitised guinea pigs. The cumulative addition of ovalbumin (0.001–100 µg/ml) led to concentration-dependent contractile responses with a *p*EC_50_ of 6.08±0.20 and a maximal effect (E_max_) of 97.1±8.5% (mean ± S.E.M) (n = 6). Pretreatment with mangiferin (0.1–10 µM) for 15 min prior to the addition of cumulative concentrations of ovalbumin inhibited anaphylactic contractions in a concentration-dependent manner (Figure 1A). Furthermore, mangiferin (0.1 µM, 1 µM and 10 µM) affected both the potency and maximal response to ovalbumin, causing rightward shifts of 2-, 22- and 562-fold, respectively (Table 1). Next, tracheal rings from naïve guinea pigs were used to demonstrate that the contraction elicited by cumulative concentrations of distinct spasmogens, such as histamine (Figures 1B), 5-HT (Figure 1C) or carbachol (Figure 1D), were also inhibited by mangiferin in a concentration-dependent manner. As shown in Table 1, 1 µM and 10 µM mangiferin caused rightward shifts of 3.8- and 67.6-fold for histamine, 12.3- and 51.3-fold for 5-HT and 1.4- and 4-fold for carbachol, respectively. At these concentrations, mangiferin also reduced the maximal response to histamine (from 111±4% (n = 7) to 90±2% (*p*<0.01, n = 6) and 65±4% (*p*<0.001, n = 5)), 5-HT (from 83±2% (n = 5) to 46±3% (*p*<0.01, n = 6) and 30±6% (*p*<0.001, n = 8)) and carbachol (from 137±2% (n = 6) to 111±4% (n = 7) and 79±5% (*p*<0.001, n = 7)).

**Table 1:** Potency (*p*EC_50_) and maximal response (E_MAX_) values obtained from concentration-response curves of allergen (ovalbumin, 0.001–100 µg/ml), histamine (0.1–3000 µM), 5-HT (0.01–30 µM) or carbachol challenge (0.01–100 µM) in guinea pig tracheal rings, following co-incubation with mangiferin (0.1–10 µM) or vehicle (0.1% DMSO).

Mangiferin (µM)	Ovalbumin		Histamine		5-HT		Carbachol	
	*p*EC_50_	E_MAX_ (%)	*p*EC_50_	E_MAX_ (%)	*p*EC_50_	E_MAX_ (%)	*p*EC_50_	E_MAX_ (%)
0 (vehicle)	6.08±0.20	97.1±8.5	5.53±0.12	111.3±4.3	6.15±0.14	82.9±2.1	7.12±0.06	137.1±2.1
0.1	5.78±0.13	75.1±6.2	5.11±0.10*	101.9±2.6	5.93±0.04	74.8±2.5	7.13±0.09	129.7±2.5
1.0	4.73±0.44*	51.6±7.6**	4.95±0.10**	89.7±2.4**	5.06±0.17**	46.4±2.8	6.97±0.06	110.9±3.7
10.0	3.33±0.09***	17.4±2.6***	3.70±0.34***	65.2±3.9***	4.44±0.19**	30.3±5.7	6.52±0.20*	79.1±5.3***

Data represent the mean ± S.E.M. of 4 to 8 tracheal segments.

* *p*<0.05,

** *p*<0.01 and.

*** *p*<0.001 compared with the tracheal responses of vehicle-treated tissues.

### Epithelial Removal Impairs the Anti-Spasmodic Effect of Mangiferin

**Figure 2:**
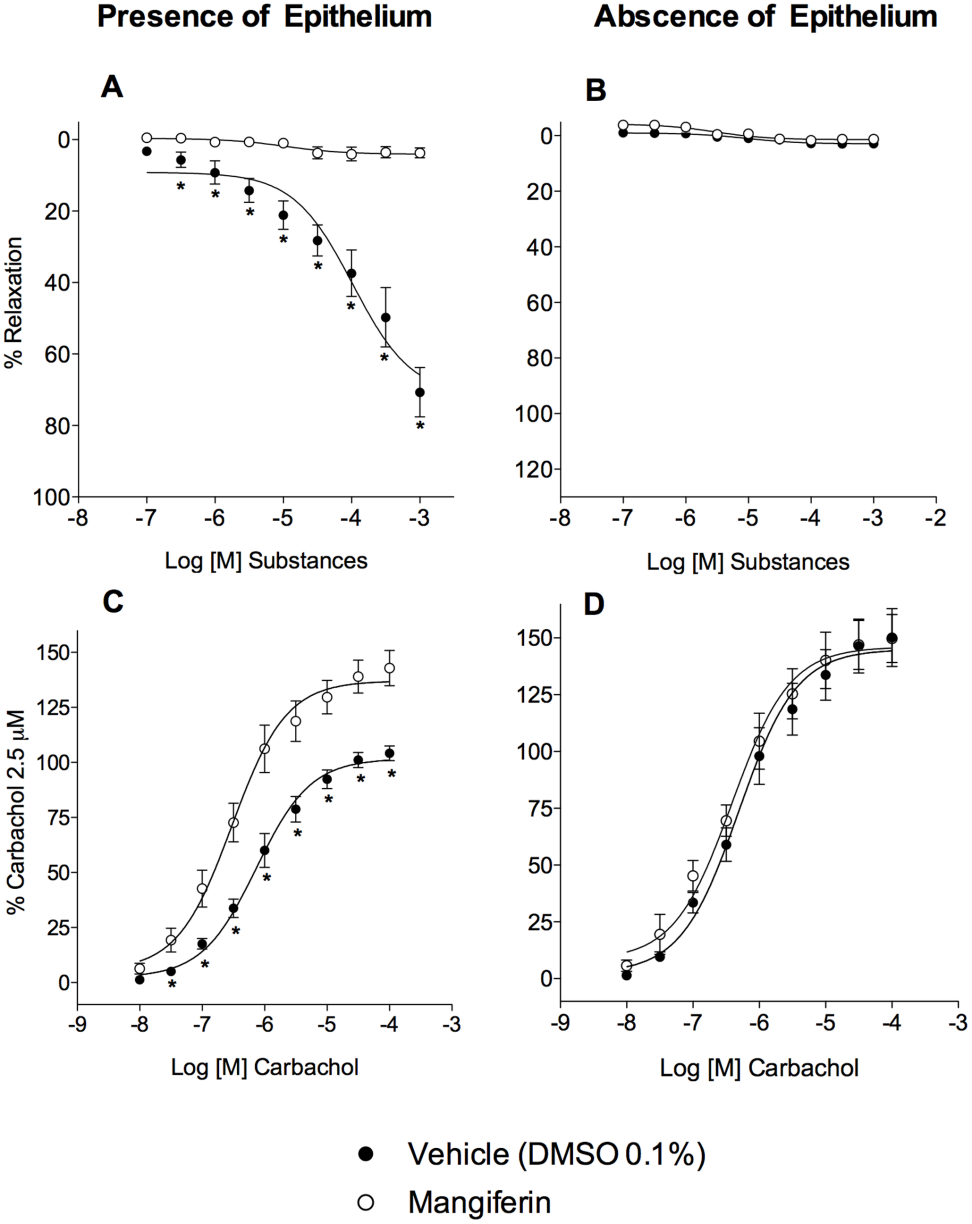
Involvement of the epithelium in the protective effect of mangiferin. Tracheal relaxations induced by cumulative concentrations of mangiferin in guinea-pig trachea that were precontracted with 2.5 µM carbachol in the presence (A) or absence (B) of epithelium. The antispasmodic effect of 10 µM mangiferin on tracheal contractions induced by carbachol (0.01–100 µM) in the presence (C) or absence (D) of epithelium. Each point represents the mean ± S.E.M. of 5 to 6 segments. All results are expressed as a percentage of the contractile response induced by 2.5 µM carbachol. *p<0.05 compared with the tracheal responses of vehicle-treated tissues (open circles).

Next, we examined whether the epithelium is involved in the effect of mangiferin by mechanically removing the epithelial cells from the internal tracheal surface with a fine silver wire, as previously reported [31]. Histological examination confirmed that the epithelial layer was removed (data not shown). The relaxant effect of mangiferin on carbachol pre-contracted tracheal segments (Figure 2A) was abolished following epithelial removal (Figure 2B). Similarly, the ability of mangiferin to impair carbachol-induced contraction (Figure 2C) was abolished after epithelial denudation (Figure 2D).

### Inhibition of NOS or Guanylate Cyclase Prevents the Anti-Spasmodic Effect of Mangiferin

**Figure 3:**
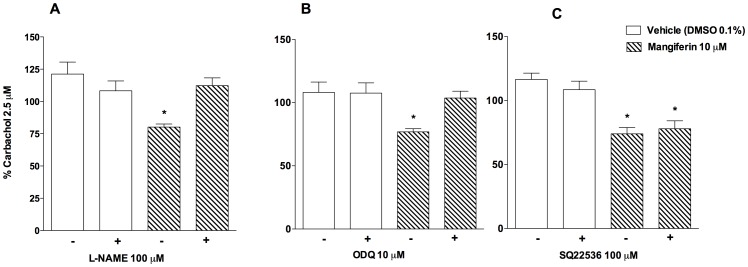
Involvement of nitric oxide in the antispasmodic effect of mangiferin. Effects of mangiferin (10 µM) on carbachol-contracted guinea pig trachea, performed in absence or presence of L-NAME (A), ODQ (B), or SQ22536 (C). Each point represents the mean ± S.E.M. of 6 segments. All results are expressed as a percentage of the contractile response induced by 2.5 µM carbachol. *p<0.05 compared with tracheal responses of vehicle-treated tissues.

Tracheal rings were pretreated with the NOS inhibitor, L-NAME (100 µM), to examine the role of nitric oxide-mediated signalling in the antispasmodic effect of mangiferin. As shown in figure 3A, L-NAME prevented the relaxing effect of mangiferin after carbachol-induced tracheal contraction. This response was also abrogated by pre-incubation with the soluble guanylate cyclase inhibitor, ODQ (10 µM) (Figure 3B), whereas pretreatment with the adenylate cyclase inhibitor, SQ22536 (100 µM), did not alter the protective effect of mangiferin (Figure 3C).

### Mangiferin Increases NOS3 Expression in Cultured Tracheal Rings

**Figure 4:**
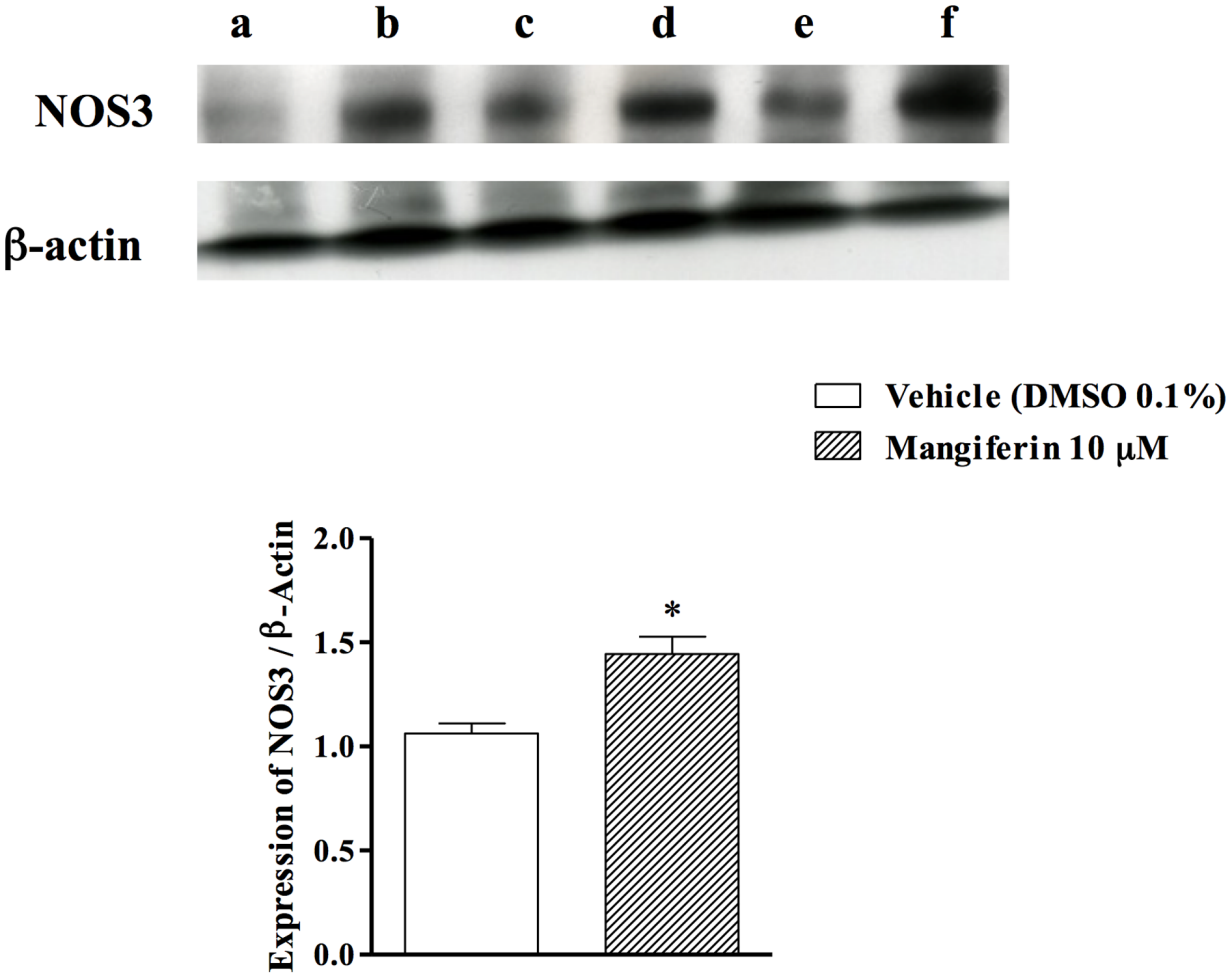
Mangiferin-induced up-regulation of NOS3 in cultured tracheas. Expression of NOS3 in rat tracheas was determined by Western blot. a, c and e are lanes of trachea pools incubated with vehicle, while b, d and e are lanes of trachea pools incubated with mangiferin for 15 min. Data were normalized to β-actin and represented as the ratio between expression of NOS3:β-actin. Each bar represents the mean ± S.E.M. of 3 pools containing 3 tracheas each. * *p*<0.05 as compared with vehicle-treated groups.

To determine whether NOS3 isoform was up-regulated by mangiferin treatment, western blotting analyses were performed on tracheal tissue homogenates. We found that constitutive NOS3 expression was significantly increased in cultured tracheal rings exposed to 10 µM mangiferin, as compared to vehicle exposed rings (Figure 4).

### Mangiferin Increases cGMP Levels in Cultured Tracheal Rings

**Figure 5:**
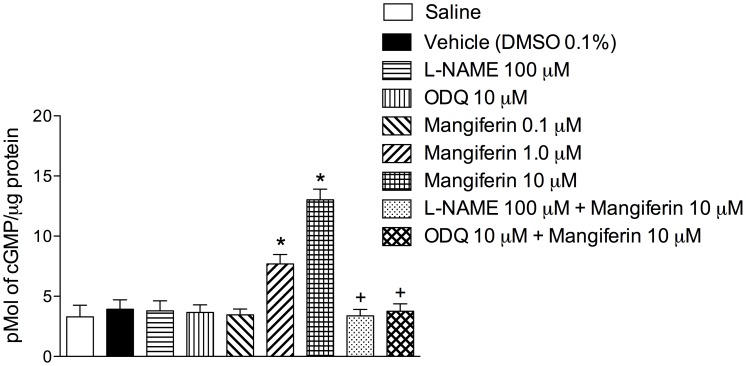
Mangiferin increases cGMP levels in cultured tracheal rings. Effect of mangiferin on tracheal cGMP levels in the presence or absence of L-NAME or ODQ. Each bar represents the mean ± S.E.M. of 4 segments.*p<0.05 compared with vehicle-treated tissues. +*p*<0.05 compared with 10 µM mangiferin-treated tissues.

Next, we demonstrated that mangiferin (0.1–10 µM) up-regulated the levels of cGMP in cultured guinea pig tracheal rings in a concentration-dependent manner (Figure 5). The ability of mangiferin to elevate cGMP levels was abolished by pre-incubation with either 100 µM L-NAME or 10 µM ODQ (Figure 5).

### K^+^ Channel Blockers Reduce the Antispasmodic Effect of Mangiferin

**Figure 6:**
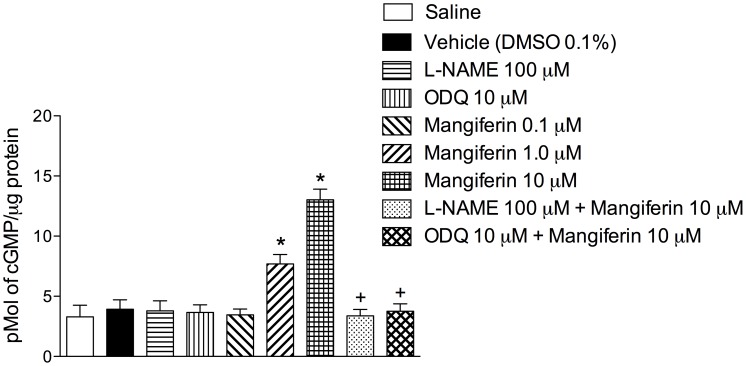
Involvement of K^+^ channels in the antispasmodic effect of mangiferin. Effects of mangiferin (10 µM) on carbachol-contracted guinea pig trachea, performed in the presence or absence of TEA (A), glibenclamide (B) or apamin (C). Each point represents the mean ± S.E.M. of 6 segments. All results are expressed as a percentage of the contractile response induced by 2.5 µM carbachol. *p<0.05 compared with vehicle-treated tissues.

Prior studies revealed that the opening of K^+^ channels leads to K^+^ efflux, hyperpolarisation and relaxation of respiratory smooth muscle [32]. The nonselective K^+^ channel blocker TEA (10 µM), the K^+^_ATP_ channel blocker glibenclamide (1 µM) and the small-conductance Ca^2+^-activated K^+^ channel blocker apamin (1 µM) were utilised to assess the potential role of K^+^ channels in the antispasmodic activity of mangiferin. The ability of individual K^+^ channel blockers to interfere with the protective effect of 10 µM mangiferin was evaluated in epithelia-preserved guinea pig tracheas that were stimulated with 2.5 µM carbachol. We found that a 15-min preincubation with TEA (Figure 6A), glibenclamide (Figure 6B) or apamin (Figure 6C), prior to the application of mangiferin, prevented the antispasmodic effect of this compound.

### Mangiferin Inhibits Ca^+2^-Induced Contraction in K^+^-Depolarised Trachea Rings

**Figure 7:**
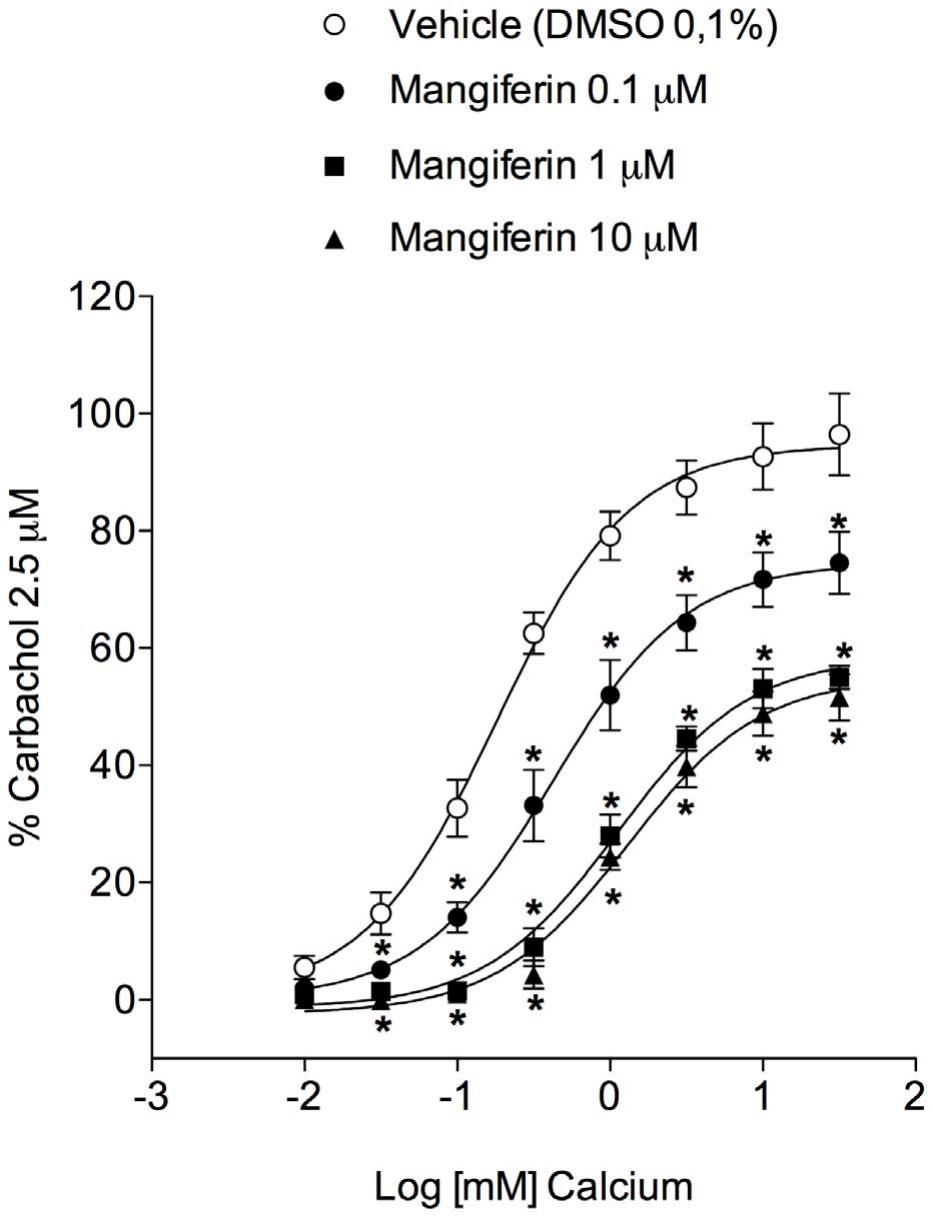
Involvement of Voltage-dependent calcium channels in the protective effect of mangiferin. Effect of mangiferin (0.01–10 µM) on the contractile response triggered after the cumulative application of calcium to tracheal rings mounted in calcium-free high K^+^ (60 mM) Krebs’ solution. Each point represents the mean ± S.E.M. from 6 segments. The magnitude of the contractile tension induced by calcium on tracheal preparations is expressed as a percentage of the contractile response evoked by 2.5 µM carbachol. *p<0.05 compared with vehicle-treated tissues.

Airway smooth muscle contraction is, in large part, regulated by intracellular Ca^2+^. Thus, we wanted to assess the effect of mangiferin on extracellular Ca^2+^-induced tracheal tension using the classical system of isolated organ bath preparations maintained in Ca^2+^-free medium and depolarised with 60 mM KCl [33]. As expected, when the extracellular Ca^2+^ concentration was increased stepwise by the cumulative addition of CaCl_2_ (0.01–30 mM), we observed a concentration-dependent elevation in the tracheal contractile response (Figure 7). As illustrated in Figure 7, preincubation for 15 min with mangiferin (0.1–10 µM) dramatically reduced Ca^+2^–induced tracheal contraction.

## Discussion

The current study shows that the xanthone glucoside mangiferin prevents the contraction of guinea pig tracheal rings induced by distinct spasmogens, including carbachol and allergen stimuli. This effect was abrogated by removal of the epithelium and exposure to the NOS inhibitor, L-NAME, or the guanylate cyclase inhibitor, ODQ. Mangiferin up-regulated NOS3 protein levels in the tracheal tissue. It also caused a dose-dependent increase in the intracellular cGMP levels of cultured tracheal rings via a mechanism that was also sensitive to L-NAME and ODQ treatments. These data suggest that mangiferin activates the nitric oxide-cGMP pathway to prevent airway smooth muscle contraction.

A previous study from Alvarez and collaborators [12] showed that asthma patients benefit from oral therapy with Vimang®, the brand name of an aqueous extract of *M. indica* stem bark that is traditionally used in the Caribbean region to treat respiratory disorders [1], [34], [35]. Accordingly, there are consistent reports demonstrating the efficacy of Vimang® and mangiferin, its prominent active ingredient, in preventing inflammatory changes in murine models of allergy and asthma [21], [36]. Interestingly, the *M. indica* extract is also a potent inhibitor of histamine- and acetylcholine-induced rat tracheal contraction in *in vitro* settings [28], [29]. Therefore, there is evidence that mangiferin has combined anti-inflammatory and airway-relaxing properties, which greatly increases the likelihood that this compound represents a potential therapeutic agent for the treatment of asthmatic conditions. To the best of our knowledge, this is the first study designed to assess the antispasmodic activity of mangiferin on the airway smooth muscle system and the first to indicate its molecular mechanism of action. Our findings revealed that mangiferin not only reduced the maximal tracheal contraction induced by distinct spasmogenic agents (allergen, histamine, carbachol and 5-HT) but also shifted the spasmogens-induced concentration-response curves to the right, clearly impacting the efficacy and potency of these agents. Moreover, it is clear that the effect of mangiferin on airway smooth muscle contractile responses is not specific for a certain type of receptor.

The observation that tracheal epithelial denudation abrogated the relaxant effect of mangiferin on carbachol pre-contracted or post-contracted segments is of great importance, and it indicates that the integrity of the airway epithelial layer is essential for the antispasmodic activity of mangiferin. Epithelial cells play a paramount role in the modulation of airway tone by working as a physical barrier that protects sensory nerves and smooth muscle cells from inhaled irritants [37]. In addition, the epithelial layer has the ability to release smooth muscle relaxant factors, such as prostaglandin (PG) E2 and nitric oxide, protecting the airway from excessive bronchoconstriction [37].

Nitric oxide activates guanylate cyclase, which increases the level of intracellular cGMP, relaxing the airway smooth muscle [38]. We preincubated isolated tracheal rings with either L-NAME or ODQ to determine the putative involvement of the nitric oxide-mediated signalling pathway for the inhibitory effect of mangiferin. Our findings revealed that treatment with both these agents prevented the anti-spasmodic activity of mangiferin, whereas the adenylate cyclase inhibitor SQ22536 had no effect. Because PGE_2_ increases airway caliber via the activation of adenylate cyclase and subsequent elevation of intracellular cyclic AMP levels (cAMP), our findings indicated that the antispasmodic effect of mangiferin is mediated by cGMP, but not cAMP, ruling out the involvement of PGE_2_ in this effect. We also measured cGMP levels in tracheal tissues exposed to mangiferin to examine whether the relaxant effect of this xanthone correlates with the accumulation of intracellular cGMP. Indeed, mangiferin raised intracellular cGMP levels in a concentration-dependent manner, and this augmentation was completely inhibited by incubation with either L-NAME or OQD. These results support the interpretation that the relaxant effect of mangiferin is dependent on the activation of the nitric oxide/cGMP signalling pathway in the airway epithelium.

*M. indica* extract (Vimang), but not mangiferin, has been shown to inhibit vascular smooth muscle contraction triggered by noradrenaline, suggesting that different polyphenols present in the extract might account for the Vimang’s vasodepressor effect [39]. However, it is of note that both Vimang and mangiferin share the ability to inhibit interleukin-1β-induced expression of inducible nitric oxide synthase (iNOS or NOS2) in vascular smooth muscle cells from WKY rats [39]. Accordingly, mangiferin and *M. indica* extract inhibit NOS2 mRNA expression and nitric oxide production in LPS-activated macrophages [40]. It is well established that despite being continuously expressed, the constitutive forms of NOS (nNOS or NOS1 and eNOS or NOS3) are also sensitive to expressional up regulation, leading to both physiological and pathophysiological consequences [41]. For instance, aminoguanidine inhibition of NOS2 activity ameliorates cerebral vasospasm after subarachnoid haemorrhage in rabbits via increase of NOS1 mRNA and protein levels [42]. While exploring the fact that respiratory epithelial cells do express NOS3 [43], we demonstrated in the current study that the treatment with mangiferin effectively increased the protein levels of this constitutive form NOS in cultured epithelium-intact tracheal tissues. These findings add support to the interpretation that mangiferin relaxant properties are closely related with activation of the nitric oxide-formation system.

The relationship between increased tissue levels of cGMP and tracheal smooth muscle relaxation in guinea pigs and other animal species has been reported [44]–[46]. Most of the nitric oxide- and cGMP-regulated signalling pathways responsible for airway smooth muscle relaxation is mediated by the opening of K^+^ channels, including small-conductance Ca^2+^-activated K^+^ and K^+^_ATP_ channels. The activation of K^+^ channels causes potassium ion efflux, plasma membrane hyperpolarisation, increased closure of voltage-gated calcium channels and, eventually, a decrease in intracellular calcium levels in smooth muscle cells [32], [47]. In the current study, we demonstrated that the anti-spasmodic property of mangiferin is lost in the presence of the non-selective K^+^ channel blocker TEA, suggesting the pivotal involvement of K^+^ channels in this response. It is also noteworthy that glibenclamide, a K^+^_ATP_ channel blocker, and apamin, a blocker of small conductance Ca^2+^-activated K^+^ channels, significantly inhibited the anti-contraction effect of mangiferin. We chose these inhibitors because K^+^_ATP_ channels and small conductance Ca^2+^-activated K^+^ channels have been strongly implicated in the airway smooth muscle relaxant response. These results suggest that both small conductance K^+^_ca_ and K^+^_ATP_ channels play a role in the inhibitory effect of mangiferin on tracheal contraction.

**Figure 8:**
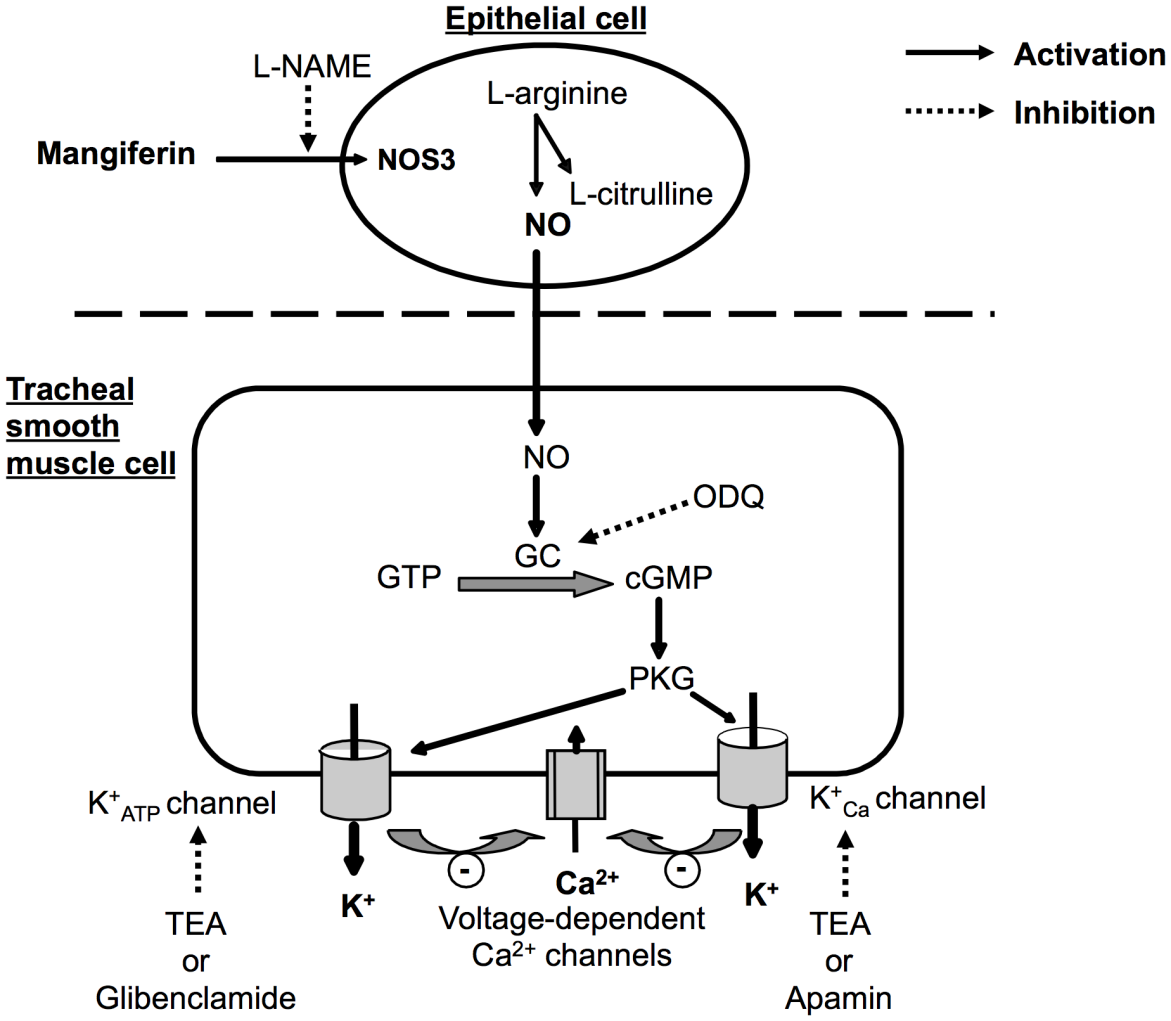
Proposed mechanism of action of mangiferin on guinea pig tracheal epithelium and smooth muscle cells. Mangiferin activates nitric oxide synthase 3 (NOS3) isoform that up-regulates the production of nitric oxide (NO) in the epithelial cell. Nitric oxide activates guanylate cyclase (GC), which increases the level of intracellular cyclic GMP (cGMP). Increased cGMP then activates the protein kinase G (PKG) cascade, enhancing K^+^ efflux and attenuating Ca^2+^ influx-associated smooth muscle cell contractility. GTP (guanosine triphosphate); L-NAME (N-nitro-L-arginine methyl ester); ODQ (1H-[1], [2], [4]oxadiazolo[4,3-a]quinoxalin-1-one); TEA (tetraethylammonium).

There is a significant body of evidence for the existence of voltage-dependent Ca^2+^ channels in airway smooth muscle [48], [49]. In the guinea pig airway, tracheal contraction evoked by KCl is induced by membrane depolarisation and the influx of Ca^2+^ through voltage-dependent Ca^2+^ channels [50]. Mangiferin also inhibited Ca^2+^-induced contractions in K^+^ depolarised preparations of epithelium-intact tracheal rings, suggesting that mangiferin could inhibit Ca^2+^ influx by blocking voltage-dependent Ca^2+^ channels. Taken together, our results indicate that the effect of mangiferin on tracheal tissue is mediated by activation of the nitric oxide cGMP pathway, leading to enhanced K^+^ efflux with subsequent attenuation of Ca^2+^ influx-associated contractility in smooth muscle cells (Figure 8).

### Conclusion

Our findings emphasise the ability of mangiferin to inhibit smooth muscle spasms caused by immunological (allergen) and non-immunological (histamine, carbachol and 5-HT) stimuli. These effects seem to be strongly associated with increased NOS3 protein levels and nitric oxide production by epithelial cells, up-regulation of intracellular cGMP and the opening of K^+^_ATP_ and small-conductance Ca^2+^-activated K^+^ channels in smooth muscle cells. These changes block voltage-dependent Ca^2+^ channels, resulting in smooth muscle relaxation. Taken together, these findings demonstrate that mangiferin may be beneficial for the treatment of airflow limitation in human lung diseases.
