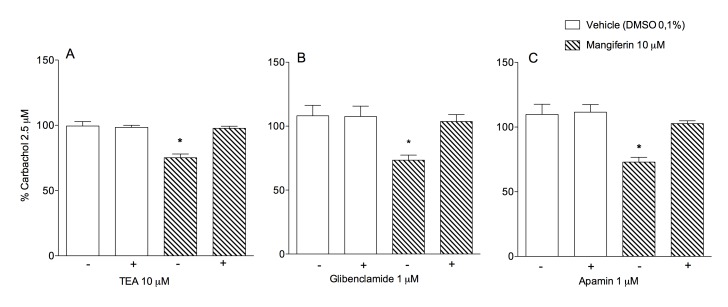# Correction: Mangiferin Prevents Guinea Pig Tracheal Contraction via Activation of the Nitric Oxide-Cyclic GMP Pathway

**DOI:** 10.1371/annotation/48d07c62-084f-4c46-b8cf-564ff59f1bd4

**Published:** 2013-09-10

**Authors:** Aline B. Vieira, Luciana P. Coelho, Daniella B. R. Insuela, Vinicius F. Carvalho, Marcelo H. dos Santos, Patricia MR. Silva, Marco A. Martins

The published Figure 6 is an erroneous duplicate of Figure 5. Please view the correct Figure 6 here: 

**Figure:**